# GenoDup Pipeline: a tool to detect genome duplication using the dS-based method

**DOI:** 10.7717/peerj.6303

**Published:** 2019-01-23

**Authors:** Yafei Mao

**Affiliations:** Marine Genomics Unit, Okinawa Institute of Science and Technology Graduate University, Onna, Okinawa, Japan

**Keywords:** Polyploidy, Whole-genome duplication (WGD), dS-based, Next generation sequencing (NGS), Software, Age distribution, GenoDup

## Abstract

Understanding whole genome duplication (WGD), or polyploidy, is fundamental to investigating the origin and diversification of organisms in evolutionary biology. The wealth of genomic data generated by next generation sequencing (NGS) has resulted in an urgent need for handy and accurate tools to detect WGD. Here, I present a useful and user-friendly pipeline called GenoDup for inferring WGD using the dS-based method. I have successfully applied GenoDup to identify WGD in empirical data from both plants and animals. The GenoDup Pipeline provides a reliable and useful tool to infer WGD from NGS data.

## Introduction

Whole (large-scale)-genome duplication (WGD), or polyploidy, has been regarded as an evolutionary landmark in the origin and diversification of animals, plants, and other evolutionary lineages (Van De Peer, Mizrachi & Marchal, 2017). Previous studies have shown that WGD plays an important role in enhancing speciation and reducing risks of extinction. Moreover, evolutionary novelty can be generated by duplicated genes via subfunctionalization, neofunctionalization, and dosage effects under WGD (Glasauer & Neuhauss, 2014; Van De Peer, Mizrachi & Marchal, 2017). Therefore, identification of WGD is the first step to understanding the impacts of WGD and the fates of duplicated genes. WGD is now known to be a common event in plants, since the availability of genomic data generated by next generation sequencing (NGS) (Jiao, 2018; Jiao & Paterson, 2014; Soltis et al., 2009; Soltis et al., 2015). Meanwhile, recent studies also suggest that WGD is a common evolutionary force in animals (Li et al., 2018; Van De Peer, Mizrachi & Marchal, 2017). Hence, an easy-to-use pipeline is urgently needed to infer WGD using NGS data.

There are three main approaches to infer WGD with NGS data (Tiley, Ane & Burleigh, 2016). First, identification of synteny blocks is the most straightforward method to detect WGD, but it requires high-quality genome assembly, and sadly, many genomes have not yet reached that assembly quality (Bowers et al., 2003). Second, phylogenetic analysis of gene families can unravel WGD when organisms have undergone extensive gene loss or genome shuffling (Jiao et al., 2011; Jiao et al., 2014), but the uncertainty of gene tree reconstruction is a serious limitation as well as heavy computation is required. Finally, analysis of rates of synonymous substitutions per synonymous site (dS) of duplicated genes (the dS-based method or age distribution method) is the most common and widely used approach to infer WGD (Lynch & Conery, 2000; Tiley, Barker & Gordon Burleigh, 2018).

Synonymous substitutions are usually under little selection, thus, rates of synonymous substitutions per synonymous site (dS) between two genes can be regarded as a proxy for the time of their divergence (Lynch & Conery, 2000). In addition, the process of gene duplication and loss is assumed as a steady birth and death model. Therefore, the distribution of dS values for paralogs should be an “L-shape” curve (Maere et al., 2005). WGD usually generates numerous paralogs simultaneously and thus a peak in the distribution of dS values can be considered as a WGD event. Compared to phylogenomic and synteny block approaches, assembled genome information and heavy computation are not required for the dS-based method. Yet because of the effects of synonymous substitution saturation and gene retention rates, it is difficult for the ds-based method to infer WGD events, which are too ancient or under lower gene retention rates (Tiley, Barker & Gordon Burleigh, 2018; Van De Peer, Mizrachi & Marchal, 2017). Despite all that, the dS-based method is still a relatively quick and easy way to infer WGD as the first step, and then the inferred WGD could be confirmed by phylogenomic and synteny block approaches.

The dS-based method is a fragmented step-wise process (Vanneste et al., 2014). Multiple software packages are required to build gene pairs, align sequences, and calculate dS values. Usually, there are two ways to build gene pairs using the dS-based method. The first one is to use paralogous gene pairs, generated by gene family cluster (orthogroup) information or gene pair information. Gene pair information is usually created by all-against-all BLAST directly while orthogroup information can be generated by a clustering algorithm based on all-against-all BLAST result (e.g., OrthoMCL Li, Stoeckert Jr & Roos, 2003, OrthoFinder Emms & Kelly, 2015). Generally, orthogroups provide more accurate information of duplicated genes rather than gene pairs. Secondly, paralogs located at the same synteny block are considered as anchor gene pairs. Thus, we could use synteny information to generate anchor gene pairs. Anchor gene pairs accurately represent duplicated genes and usually provide more information about ancient duplication events. Together, consistent results from both approaches yield a credible conclusion for the dS-based method.

DupPipe is a web-based method to infer WGD using the dS-based method (Barker et al., 2010). In addition, FASTKs is a pipeline to calculate dS values for gene pairs (McKain et al., 2016). Both DupPipe and FASTKs calculate dS values based on gene pair information, but not based on orthogroup information, to infer WGD. Here, an open-source script called GenoDup Pipeline is developed to infer WGD using the dS-based method based on orthogroup or/and (paralogous or anchor) gene pair information.

## Materials & Methods

### GenoDup Pipeline architecture

**Figure 1:**
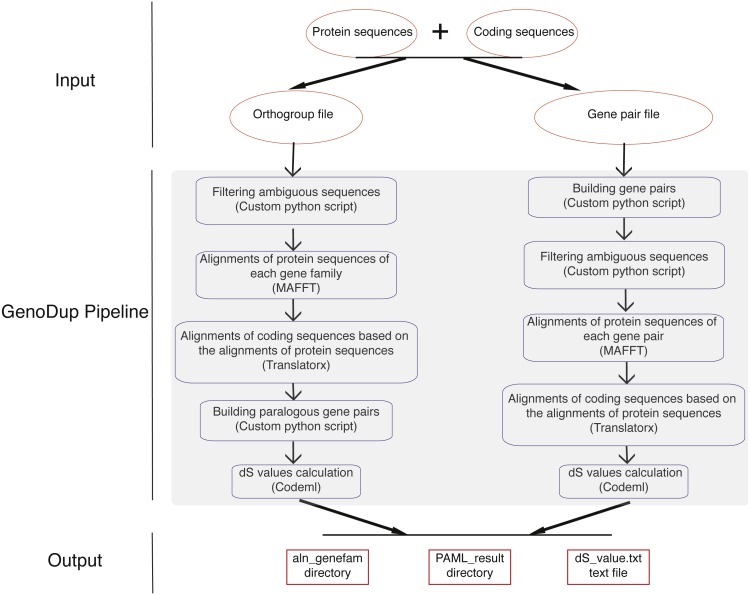
Workflow in GenoDup Pipeline. Orange oval boxes represent inputs. Blue boxes represent three subroutines in the GenoDup Pipeline. Red boxes represent outputs generated by GenoDup Pipeline.

GenoDup Pipeline is written in Python integrating with alignment of sequences, building gene pairs, and dS value calculations. BioPython must be installed and three more executable dependencies are needed: MAFFT (Katoh et al., 2002), Translatorx (Abascal, Zardoya & Telford, 2010), and Codeml package in PAML (Yang, 2007). Nuclear protein coding sequences (CDS) and the corresponding protein sequences are mandatory inputs to run GenoDup Pipeline. In addition, orthogroup information or gene pair information is another mandatory input for orthogroup and gene pair approach, respectively. Once mandatory files have been inputted appropriately, 3 subroutines in GenoDup run as follows (Fig. 1).

(1) Alignment of gene pairs: GenoDup Pipeline can automatically align gene pairs or gene families using MAFFT and Translatorx. Before performing gene pair or gene family alignments, GenoDup firstly filters ambiguous sequences that contain ‘N’, and removes CDS that mis-match to the corresponding protein sequences. Then, MAFFT is used to perform alignments of protein sequences with parameters: localpair and maxiterate: 1000; and Translatorx is used to align CDS based on alignments of the corresponding protein sequences.

(2) Building gene pairs: there are two ways to build gene pairs in GenoDup Pipeline. The first way requires an orthogroup information file and a number (N) as inputs. Only the orthogroups, which contain less than N genes, can be used to build gene pairs. GenoDup Pipeline builds n(n-1)/2 paralogous gene pairs within a gene family (n is the number of genes in a gene family). OrthoMCL is recommended to generate orthogroup (Li, Stoeckert Jr & Roos, 2003). The second way requires a gene pair information file. Paralogous gene pairs can be generated by all-against-all BLAST. Or, MCScanX (Wang et al., 2012) and i-ADHoRe (Proost et al., 2011) can be used to generate anchor gene pairs.

(3) dS value calculations: based on alignments of CDS, GenoDup can automatically build a control file, required by Codeml, for each gene pair. Codeml package in PAML is used to calculate dS values with parameters: noisy = 9, verbose = 1, runmode = −2, seqtype = 1, CodonFreq = 2, model = 0, NSsites = 0, icode = 0, fix_kappa = 0, kappa = 1, fix_omega = 0, and omega = 0.5.

All of the three subroutines above can automatically run in the GenoDup Pipeline, and finally generate two directories and a text file as outputs. One directory called aln_genefam contains all CDS alignments in Fasta format. The other directory, called PAML_result, contains all results generated by Codeml. A text file called dS_value.txt contains all dS values of gene pairs. An R script (plot_GenoDup.r) is also provided to plot dS distributions.

## Results

### Empirical data validation

To evaluate the performance of the GenoDup Pipeline, I applied it to two empirical data: one is a model plant (*Arabidopsis thaliana*) and the other is a model animal (*Oncorhynchus mykiss*). *Arabidopsis thaliana* has undergone two independent WGDs (alpha and beta WGD) and *Oncorhynchus mykiss* has experienced four independent WGDs (Ss4R, Ts3R, and Two-rounds WGD) (Berthelot et al., 2014; Vanneste et al., 2014).

**Figure 2:**
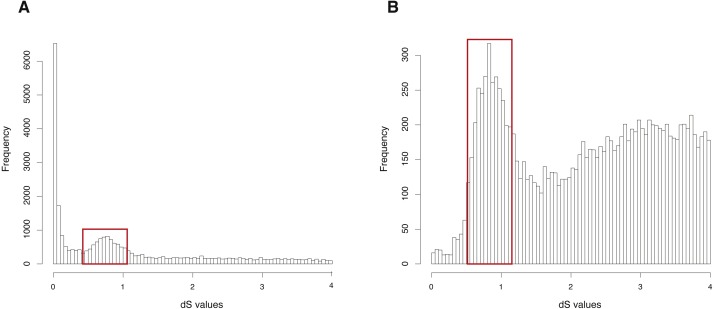
dS distributions of paralogous gene pairs and anchor gene pairs in *Arabidopsis thaliana*. (A) The peak (dS value range: 0.5∼1) marked with a red box represents a signal as alpha WGD in the dS distributions of paralogous gene pairs generated by orthogroups. (B) The peak (dS value range: 0.5∼1) marked with a red box represents a signal as alpha WGD in the dS distributions of anchor gene pairs.

The CDS, protein sequences, and genome annotation files of *Arabidopsis thaliana* were downloaded from Ensembl Plants (http://plants.ensembl.or/Arabidopsis_thaliana/Info/Index). Orthogroup information was generated with OrthoMCL and 48,307 genes of *Arabidopsis thaliana* were clustered into 5,962 orthogroups. N was set as 15, meaning that the orthogroups containing less than 15 genes were used to build gene pairs, and 68,231 paralogous gene pairs were generated in total. The entire analysis ran in 8 .8 h with 4 cores. The result showed a clearly visible peak (dS value range: 0.5∼1) in the dS distribution of paralogous gene pairs (Fig. 2A). On the other hand, MCScanX was used to generate 99,309 anchor gene pairs and the entire analysis ran in 76.8 h with 4 cores (Table 1). The result showed the same peak (dS value range: 0.5∼1) in the dS distribution of anchor gene pairs (Fig. 2B). Based on assumptions of the dS-based method, the peak represents a WGD event and the Genodup Pipeline properly detected a WGD event (alpha WGD) in *Arabidopsis thaliana.*

**Table 1:** Statistics of empirical data validation in GenoDup Pipeline.

	*Arabidopsis thaliana*		*Oncorhynchus mykiss*	
	Orthogroup	Anchor gene pairs	Orthogroup	Anchor gene pairs
The number of gene pairs	68,231	99,309	42,888	26,880
Running Time (h)^*^	8.88	76.8	7.63	17.65

**Notes.**

* The running time includes the OrthoMCL running, each of run of OrthoMCL is less than 10 min.

**Figure 3:**
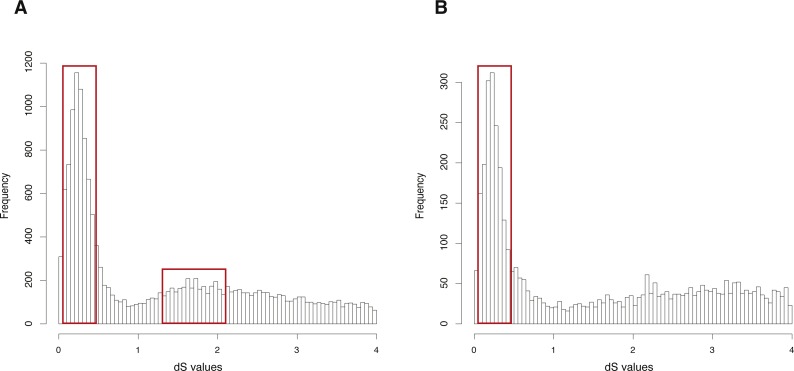
dS distributions of paralogous gene pairs and anchor gene pairs in *Oncorhynchus mykiss*. (A) The peaks (dS value ranges: 0.1∼0.5 and 1.2∼2) marked with red boxes represent signals of the Ss4R and Ts3R WGD, respectively, in the dS distributions of paralogous gene pairs generated by orthogroups. (B) The peak (dS value range: 0.1 0.5) marked with a red box represents the signal as the Ss4R WGD of Oncorhynchus mykiss in the dS distributions of anchor gene pairs.

The CDS, protein sequences, and genome annotation files of *Oncorhynchus mykiss* were downloaded from GENEOSCOPE (http://www.genoscope.cns.fr/trout/data/). Orthogroup information was generated by OrthoMCL and 46,585 genes of *Oncorhynchus mykiss* were clustered into 6,562 orthogroups. N was set as 15, meaning that the orthogroups containing less than 15 genes were used to build gene pairs, and 42,888 paralogous gene pairs were generated in total. The entire analysis ran in 7.63 h with four cores. The result showed two clearly visible peaks (dS value ranges: 0.1∼0.5 and 1.2∼2) in the dS distribution of paralogous gene pairs (Fig. 3A). On the other hand, MCScanX was used to generate 26,880 anchor gene pairs and the entire analysis ran in 17.65 h with four cores (Table 1). The result showed a peak (dS value range: 0.1∼0.5) in the dS distribution of anchor gene pairs (Fig. 3B).

GenoDup Pipeline represented consistent result as the previous study on *Arabidopsis thaliana* and *Oncorhynchus mykiss*, respectively (Berthelot et al., 2014; Vanneste et al., 2014), indicating that GenoDup Pipeline is a reliable tool to infer WGD using the dS-based method.

### Comparison GenoDup with FASTKs

The dS-based method have been applied to many studies to infer WGD (Jiao et al., 2012; Vanneste et al., 2014; Zhao et al., 2017) and some software has been built to make this process easier, such as DupPipe, FASTKs, and CoGe. DupPipe and CoGe are web-based methods (Barker et al., 2010; Lyons et al., 2008), while FASTKs is an open source pipeline (McKain et al., 2016). Hence, I compared these two open source pipelines (FASTKs and GenoDup) on three datasets: one is an example set in FASTKs (*Typha angustifolia*) and other two are example sets in this study (*Arabidopsis thaliana* and *Oncorhynchus mykiss*).

**Figure 4:**
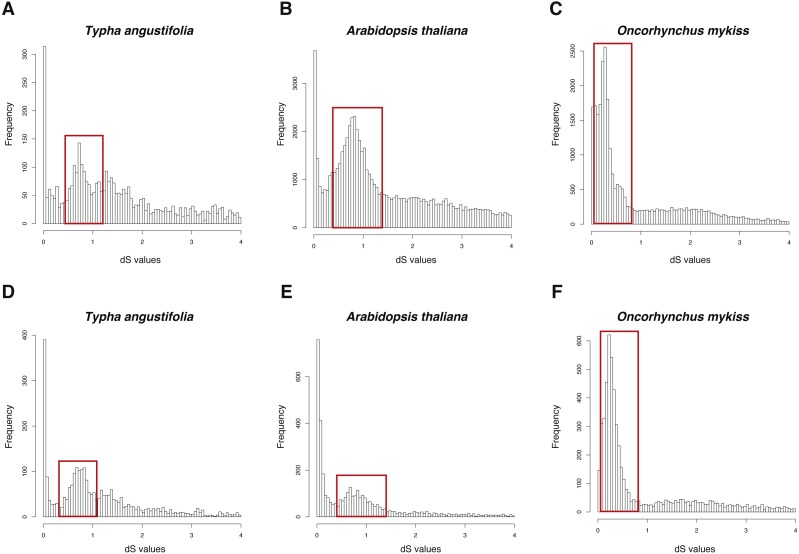
dS distributions of three datasets inferred by GenoDup and FASTKs. The dS distributions inferred by FASTKs on (A) *Typha angustifolia* (B) *Arabidopsis thaliana* (C) *Oncorhynchus mykiss*. The dS distributions inferred by GenoDup on (D) *Typha angustifolia* (E) *Arabidopsis thaliana* (F) *Oncorhynchus mykiss*. The peak marked with a red box represents a WGD event.

The three datasets were run in FASTKs with 4 cores and default settings. While, for GenoDup Pipeline, I firstly used OrthoMCL to build the orthogroups for each dataset and run GenoDup Pipeline with four cores and N was set as 5. I found that both pipelines could infer WGD events properly (Fig. 4). In addition, I found that GenoDup Pipeline used less memory rather than FASTKs in all three datasets.

## Discussion

The rapid development of NGS technologies has enabled generation of massive amounts of data, allowing us to better understand the evolutionary history of all organisms. WGD is suggested to have occurred in diverse organismal groups (Li et al., 2018; Van De Peer, Mizrachi & Marchal, 2017); thus, a reliable and efficient tool to detect WGD with NGS data is greatly needed. I developed a reliable and easy-to-use tool called GenoDup Pipeline to infer WGD using the dS-based method. GenoDup Pipeline is written in Python and can be run with one command. It is easy to use for researchers who have little experience in bioinformatics.

GenoDup Pipeline runs faster when taking orthogroup information as input rather than taking gene pair information as input (Table 1). Because the total time of alignment process is usually longer for gene pairs than for orthogroups in GenoDup Pipeline. Additionally, FASTKs runs faster for *Typha angustifolia* dataset but slower in *Arabidopsis thaliana* dataset compared with GenoDup Pipeline. One possible reason is that there are many gene pairs (123,145) generated by all-against-all BLAST in *Arabidopsis thaliana* dataset than that of *Typha angustifolia* (10,119) dataset (Table 2). In other words, the running time of FASTKs is depended on the complexity of genomes. In contrast, the running time of GenoDup Pipeline is related to the parameter N (Tables 1 and 2).

**Table 2:** Performance comparison between FASTKs and GenoDup.

	*Typha angustifolia*			*Arabidopsis thaliana*			*Oncorhynchus mykiss*		
	The number of gene pairs	Running time (h)	Maximum memory usage (Mb)	The number of gene pairs	Running time (h)	Maximum memory usage (Mb)	The numer of gene pairs	Running time (h)	Maximum memory usage (Mb)
FASTKs	10,119	0.4	417	123,145	9.5	1,066	43,704	1.8	1,263
GenoDup	6,853	0.8^*^	85	9,051	1.6^*^	145	8,910	1.2^*^	218

**Notes.**

* The running time includes the OrthoMCL running, each of run of OrthoMCL is less than 10 min.

The empirical validation shows that the analysis of *Arabidopsis thaliana* generated with GenoDup Pipeline presented a clearly visible signal for a WGD event (alpha WGD) but the signal of the second WGD event (beta WGD) was lost (Fig. 2). This result is consistent with previous studies because the dS methods cannot infer WGD when organisms have undergone extensive gene loss or genome shuffling, especially for plants (Rabier, Ta & Ane, 2014; Tiley, Ane & Burleigh, 2016). Additionally, the *Oncorhynchus mykiss* analysis with GenoDup showed two WGD signals (Ss4R and Ts3R) with orthogroup information (Fig. 3A) (Rabier, Ta & Ane, 2014). Yet, The Ts3R signal was lost in the distribution of anchor gene pairs because there were few anchor gene pairs in the analysis (Fig. 3B, Table 1). Moreover, the analyses on *Oncorhynchus mykiss* using orthogroups and anchor gene pairs did not present the two-round WGDs because the two-round WGDs are too ancient to infer with the dS-based method. Importantly, the dS-based method is widely debated for generating artificial signals, as a result of dS saturation when the dS value >1 (Vanneste, Van de Peer & Maere, 2012). In addition, the dS-based method is not useful to infer WGD on genomes which have lower gene retention rates. As well, the young WGD events are not easy to be inferred because of allelic variations (Tiley, Barker & Gordon Burleigh, 2018). Thus, GenoDup Pipeline is suitable to infer WGD when the dS value <1. However, other evidence from phylogenetic analysis and/or synteny block analysis is also needed for drawing a conclusive result.

In all, This study presents a reliable and user-friendly tool to infer WGD using the dS-based method, beneficial for large developing sequencing projects (1KP, 10KP-EBP, 1KITE, and i5K) (Li et al., 2018; Van De Peer, Mizrachi & Marchal, 2017).

## Additional Information and Declarations

### Data Availability

The following information was supplied regarding data availability: GitHub: https://github.com/MaoYafei/GenoDup-Pipeline.
